# Status of global health fellowship training in the United States and Canada

**Published:** 2019-11-28

**Authors:** Ann Evensen, Sean Duffy, Russell Dawe, Andrea Pike, Brett D. Nelson

**Affiliations:** 1 Department of Family Medicine and Community Health, University of Wisconsin School of Medicine and Public Health, Wisconsin, USA; 2 Discipline of Family Medicine, Faculty of Medicine, Memorial University of Newfoundland, Newfoundland, Canada; 3 Primary Healthcare Research Unit, Memorial University of Newfoundland, Newfoundland, Canada; 4 Divisions of Global Health and Neonatology, Department of Pediatrics, Massachusetts General Hospital, Massachusetts, USA; 5 Harvard Medical School, Massachusetts, USA

## Abstract

**Background:**

Increasing numbers of residency graduates desire global health (GH) fellowship training. However, the full extent of training options is not clear.

**Objective:**

To identify clinical GH fellowships in all specialties in the U.S. and Canada and to describe their demographics, innovative features, and challenges.

**Methods:**

The authors surveyed program directors or designees from GH fellowships with a web-based tool in 2017.

**Results:**

The authors identified 85 programs. Fifty-four programs (63.5%) responded confirming 50 fellowships. One- third of fellowships accepted graduates from more than one specialty, and the most common single-specialty programs were Emergency Medicine and Family Medicine. Fellowships most commonly were 24 months in duration with a median size of one fellow per year. Funding and lack of qualified applicants were significant challenges. Most programs were funded through fellow billing for patient care or other self-support.

**Conclusion:**

The number of U.S. and Canadian GH fellowship programs has nearly doubled since 2010. Challenges include lack of funding and qualified applicants. Further work is needed to understand how best to identify and disseminate fellowship best practices to meet the diverse needs of international partners, fellows, and the patients they serve and to determine if consensus regarding training requirements would be beneficial.

## Abstract (fr)

**Contexte:**

Un nombre croissant de diplômés des programmes de résidence optent pour une formation complémentaire en santé mondiale. Cependant, la pleine mesure des possibilités de formation n’est pas claire.

**Objectif:**

Identifier les formations cliniques complémentaires en santé mondiale pour toutes les spécialités aux États- Unis et au Canada et décrire leur démographie, leurs caractéristiques novatrices, et leurs défis.

**Méthodes:**

En 2017, les auteurs ont interrogé les directeurs de programmes de formation complémentaire en santé mondiale ou leur représentant à l’aide d’un outil en ligne.

**Résultats:**

Les auteurs ont identifié 85 programmes. 54 programmes (63,5 %) ont répondu et confirmé 50 programmes de formation. Un tiers des programmes acceptaient des diplômés provenant de plusieurs spécialités, et les programmes offerts à des spécialités uniques étaient plus fréquemment ceux en médecine d’urgence et en médecine familiale. Les programmes étaient généralement d’une durée de 24 mois avec une capacité d’accueil d’un moniteur (fellow) par année. Le financement et le manque de candidats qualifiés étaient des défis de taille. La plupart des programmes étaient financés par la rémunération des moniteurs (fellows) pour les soins qu’ils prodiguaient aux patients ou via d’autres aides financières individuelles.

**Conclusions:**

Le nombre de programmes de formation complémentaire en santé mondiale a presque doublé depuis 2010. Les défis sont notamment le manque de financement et de candidats qualifiés. Il est nécessaire de poursuivre le travail pour pouvoir bien identifier et transmettre les meilleures pratiques en matière de formation complémentaire afin de répondre aux divers besoins des partenaires internationaux, des moniteurs (fellows) et des patients qu’ils soignent, et déterminer si un consensus concernant les exigences de formation serait bénéfique.

## Introduction

Over the past four decades, interest in global health (GH) among physicians-in-training has increased dramatically.^1^^–^^3^ GH fellowships – which provide advanced training in GH beyond the clinical requirements of residency – have existed since at least 1997.^4^

As GH medical school electives, residency tracks, and fellowships become more common, it is important that trainees, program directors, international partners, and future employers understand the scope and value of these experiences. The first survey of U.S. GH fellowships documented the growing number and variety of GH fellowship opportunities available in 2010 and described program characteristics such as size, duration, specialty, and educational activities.^5^ Subsequently, profiles of individual GH fellowships^6–10^ and reviews of GH opportunities within subspecialty fellowships^11–16^ have been published. However, no subsequent studies have examined trends across all specialties.

Our objectives with this study were to identify all active U.S. and Canadian GH fellowships in all specialties and to describe their features including innovations, challenges, and graduate activities.

## Methods

A GH fellowship was defined as formal medical training beyond the usual requirements and length of residency. Fellowships that followed the completion of an accredited residency program or were integrated within a residency program (but extended its length) were included. Fellowships that were solely research-based were excluded to improve comparability amongst programs.

We identified GH fellowship programs from multiple sources, including 1) the Global Health Fellowship Database (globalhealthfellowships.org);^5^ 2) peer- reviewed and gray literatures; 3) epidemiologic snowball sampling, in which participants identified programs not currently listed in the Global Health Fellowship Database; and 4) web searches. Inclusion criteria were programs which: 1) required an additional training period beyond residency requirements, 2) self-identified as ‘global health’ or were identified as such by others through snowball recruitment, and 3) included a clinical training component.

We contacted fellowship directors or their programs’ listed point of contact using publicly-available information. Study participants completed a web- based survey (Survey Monkey, San Mateo, CA). We reminded non-respondents to complete the survey with email and, if needed, telephone reminders. We collected data from March to July 2017.

An author with expertise in survey design (AP) led the survey development. The survey contained up to 36 (using skip-logic) closed- and open-response questions (Supplementary Materials, Appendix) and was pilot-tested prior to distribution.

This study was reviewed and exempted by institutional review boards of the University of Wisconsin School of Medicine and Public Health, Massachusetts General Hospital, and by the Health Research Ethics Authority of Newfoundland and Labrador.

## Results

We identified 85 potential fellowship programs. Fifty- four programs responded (63.5%), of which 50 (92.6%) offered a GH fellowship (Supplementary Materials, Figure s1). Of the four remaining respondents, two had closed their fellowships, one never had a fellowship, and one is intending to start a fellowship. Thirty-one programs did not respond but were considered probable active fellowships based on careful review of their websites. We requested and received permission to use each program’s information such as location and contacts in the Global Health Fellowship Database (globalhealthfellowships.org). Our data reflect survey responses from the 50 confirmed fellowships unless otherwise indicated.

### Fellowship program characteristics

Table 1 lists program characteristics such as duration, location, and size. The majority of programs were located on the East Coast of the U.S. (Supplementary Materials, Figure s2).

**Table 1:** Characteristics of active fellowship programs

**Clinical specialty**	**Number of fellowship programs accepting applicants from clinical specialty(n=50)**		
**Anesthesia**	4		
**Emergency Medicine**	23		
**Family Medicine**	22		
**Internal Medicine**	12		
**Medicine-Pediatrics**	6		
**Obstetrics and gynecology**	5		
**Pediatrics**	8		
**Psychiatry**	1		
**Surgery**	3		
**Other discipline (advanced practice nursing)**	2		
**Length of program**	**Number of programs (n=50: 46 programs that follow residency training plus 4 integrated residency-fellowship programs)**		
**6 months**	1 (2.0%)		
**12 months**	17 (34.0%)		
**24 months**	26 (52.0%)		
**Other**	6 (12.0%)		
**Funding source**	**Number of programs using funding source (n=47)** ^a^		
**Fellow self-support** ^b^	45 (95.7%)		
**Department or academic institution funds**	32 (68.1%)		
**Private foundation**	13 (27.7%)		
**Graduate medical education or government**	8 (18.2%)		
**International partner**	8 (18.2%)		
**Fellowship activities**	**Number of programs requiring or offering this activity (n=46)**		
	**Mandatory**	**Optional**	**Not available**
**Clinical work**	45 (97.8%)	1 (2.2%)	0
**Coursework**	38 (82.6%)	8 (17.4%)	0
**Research**	33 (71.7%)	12 (26.1%)	1 (2.2%)
**Policy or advocacy work**	12 (28.3%)	32 (69.6%)	1 (2.2%)
**Teaching by fellow**	40 (87.0%)	6 (13.0%)	0
**Partnership organizations**	**Number of programs forming this partnership (n=46)**^a^		
**Medical schools and residencies in LMICs**	36 (78.3%)		
**Non-governmental organizations**	32 (69.6%)		
**Policy-makers/governments**	21 (45.7%)		
**Industry/private sector**	8 (17.4%)		
**Indigenous band/tribal councils**	7 (15.2%)		
**Other**	11 (23.9%)		
**None**	2 (4.3%)		

a more than one option could be chosen

b self-support includes fellow covering own expenses and/or generating revenue domestically through patient care in clinic, urgent care, hospital, or community health center

Abbreviation: LMICs = low- and middle-income countries

Coursework was primarily completed in resource-rich areas of North America (n=39, 86.7%). Research and policy/advocacy work were primarily done in resource-limited settings in low- and middle-income countries (LMICs) (research: n=42, 91.3%; policy: n=33, 82.5%). Clinical work was commonly performed in resource-rich settings in North America (n=34, 73.9%) and resource-limited settings in both North America (n=24, 52.2%) and in LMICs (n=35, 76.1%) (Supplementary Materials, Table s2).

### Fellowship program challenges and innovations

Program representatives ranked six challenges (6 = most and 1 = least significant). Mean ranking is presented here. Lack of funding (4.5) and qualified applicants (4.1) were ranked most challenging. Lack of political/institutional support (3.7), experienced GH faculty (3.6), fellowship accreditation (2.6), and international placement sites (2.5) were ranked less challenging.

Respondents could provide free-text responses for other perceived challenges and innovative or important aspects of their programs (Table 2).

**Table 2:** Examples of self-identified challenges, program changes, and important or innovative activities reported by GH fellowship programs

**Examples of challenges or program changes**	
**Funding**	• Lack of political support jeopardizes the program
**Systems**	• Balancing structure with flexibility and customization especially since essentials of GH training have yet to be formalized • Grant management and timely approval from institutional review boards • Lack of adequate clinical volume
**Applicant recruitment**	• Difficulty reaching potential applicants and tailoring to interests • Increasing number of fellowship positions creates competition • Lack of credibility of GH training; “why should I do this fellowship?”
**Field site**	• Changes in political environment (e.g., war, doctors' strike) • Lack of mutual understanding amongst partners and decision-makers regarding timeline and structure • Difficulty securing housing in low-resource environments
**Examples of innovative or important program features**	
**Structural**	• Multidisciplinary: accept physicians, registered nurses, allied health professionals, PhDs • Recruitment pairing: recruit one fellow from underserved partner site for every US-trained fellow • Trans-mentorship model for research: pairs fellows from one discipline with senior investigators from a different discipline; provides fellows with multiple sources of intellectual, practical, and career guidance • Fellow-driven program: fellows have freedom and funding to develop projects of interest • Advocacy: write policy documents and opinion pieces • Patient care opportunities: provide care in North American and international locations such as: ∘ Indigenous, migrant farmworker, or refugee health ∘ Inner-city ∘ Critical access hospital
**Education and training**	• Specialized training of fellows: ∘GH simulation^40^ ∘Faculty development ∘Ultrasound ∘Trauma-informed care ∘Humanitarian aid ∘Language ∘Burn care ∘Dentistry ∘Anesthesia ∘GH delivery • G-LOCAL experience: combined community medicine/GH fellowship • Certifications and Master’s degree programs ∘Masters in Public Health [traditional and online] ∘Masters in Science ∘Masters in Science in Clinical Investigation ∘Masters in Medical Management ∘Masters in Clinical Epidemiology and Health Services Research ∘International Diploma in Humanitarian Assistance ∘Diploma in Tropical Medicine and Hygiene
**Field site**	• Supervision: fellows work with the fellowship director in a low-resource setting the majority of the time • Contributing to host education: ∘Family Medicine residency education in LMICs, including curriculum development ∘Fellows partner with host institution on quality improvement projects and host-country continuing medical education

### Fellowship graduate characteristics

Respondents estimated that from 2012-2016 their programs each graduated a cumulative total of 0-19 graduates (median 2). Thirteen programs (26.0%) had yet to graduate a fellow so were excluded from post- fellowship analyses.

Twenty-six programs tracked their graduates’ activities through surveys, interviews, or informal contact. Graduates commonly participated in direct patient care (n=24, 92.3%), education (n=22, 84.6%), and research (n=14, 53.8%). Fewer than half of graduates participated in advocacy, policy development, or administration. Sixteen respondents provided an estimate of the proportion of their graduates working three or more months per year in LMICs (range 0-100%, mean 49.6%).

### Comparison of 2010 and 2017 fellowships

In the 2010 survey by Nelson *et al*., 80 programs in the U.S. self-identified as GH fellowships.^5^ However, residency track-only programs were not specifically excluded from that study. Because of the substantial differences in depth of training and oversight between a residency track and a fellowship program,^17^^,^^18^ we required programs to meet a more stringent definition of GH fellowship for our survey. We determined that only 39 U.S. programs in 2010 would have met our study’s definition of a GH fellowship, not 80 reported by Nelson *et al*. While Nelson, *et al* did not survey Canadian programs in 2010, three of the Canadian programs (42.9%) identified in our study were founded prior to 2010 .

## Discussion

We identified 81 total U.S. and Canadian GH fellowships, and 50 programs across various medical specialties responded to our survey. We found that lack of funding and qualified applicants were the greatest challenges for fellowship programs.

The majority of respondents in our survey (95.7%) report some type of fellow self-support as a means of funding the training program. Although complex, current fellowship billing rules provide an opportunity for sustainable global health education programs that serve domestic or (indirectly) international underserved populations. In the U.S., Accreditation Council for Graduate Medical Education (ACGME)- accredited fellowship programs (e.g., sports medicine, hospice and palliative medicine, and many others) bill for fellow services at a designated fraction of the fee charged for the same service by an attending. These programs also typically receive some funding through the U.S. government and the hospital in which the fellow is based. However, if a residency graduate joins a non-accredited fellowship (e.g., global health), the fee charged for the fellow’s service is the same as the attending physician’s fee. The fellow’s income is typically lower than the attending because fellowship programs use some of the receipts to cover expenses related to education and administration of the fellowship. This self- support funding model may make training programs more attractive to leaders, decision-makers, and communities.^19^ Detailed tracking of GH fellowship graduates is needed to understand the long-term outcomes of training and create a compelling argument for a positive return-on-investment for government funding.^20^^–^^23^

We estimate the total number of U.S. fellowship programs (according to our definition) grew from 39 in 2010 to 74 in 2017 (increase of 89.7%). This exceeds growth seen in GH training opportunities for medical students and residents.^2^^,^^3^ Out of 1,063 U.S. family medicine (FM) residents surveyed who were planning fellowship training, only 2.1% intended to apply for FM GH fellowships.^24^ Further study is warranted to determine how well fellowship opportunities match the demand for post-residency GH training. This could include subgroup analysis by specialty, region, or format/content of programs so programs struggling with vacancies could learn from subgroups that excel at recruitment.

Despite challenges, respondents described a multitude of fellowship innovations. Programs reported innovative teaching opportunities, advanced training courses, and varied settings for patient care that were consistent with best practices for international partnerships.^25^^–^^30^ Our study identified many opportunities for growth in the field of GH fellowship training such as improving interprofessional training, building partnerships with tribal councils, honing advocacy skills, and pairing fellows from high-resource and low-resource institutions. In the face of the rapid increase in GH fellowship programs and the common problems of funding and lack of qualified applicants, it is critical to continually reassess and prioritize needs of the international partners to ensure mutual benefit for all participants.

Next steps in the field of GH fellowship training should include discussion amongst U.S. and Canadian program leaders, current and potential international partners, and GH fellows to optimize fellowship structure, funding, and competencies. Preliminary work to define GH competencies at the residency and fellowship level has been published already.^31^^–^^36^ A demographic survey of fellows and *potential* fellows is needed to inform this work. Understanding factors such as ethnicity, gender, sexuality, and socio- economic background may help educators and partners prioritize competencies and overcome unintended biases that may be influencing their programs.

While our response rate was higher than typical web- based surveys,^37^^–^^39^ the actual number of fellowships could be larger if our search failed to identify programs, or smaller, if selection bias led to a greater proportion of closed programs among our 31 non- responders.

In addition to the fellow demographic study described above, future studies could characterize non-clinical, research-based programs, alternatives for physicians preparing for a career in GH (e.g., diploma or certificate programs in tropical medicine, public health, or health administration) and why some GH fellowship programs have closed. Further study of funding models and matching of high-quality fellowships sites and fellow candidates would be beneficial. Such global fellowships may want to establish a type of voluntary registry so that the data can be updated regularly and changes monitored more easily.

### Conclusion

The number of U.S. and Canadian GH fellowship programs has nearly doubled since 2010. Major challenges include lack of funding and qualified applicants. Further study is needed to assess 1) whether the quickly growing number of GH fellowships may have exceeded applicant demand, 2) how training programs can meet the needs of both international partners and a diverse group of fellows, and 3) how to incorporate and align innovations and best practices in education, research, and advocacy to ensure improved patient outcomes. Although our study did not identify any GH program accredited by the ACGME, fellowship program leaders should consider whether consensus on core competencies and minimum training requirements would be beneficial for fellows, their employers, and patients.

## Figures and Tables

**Figure s1.**
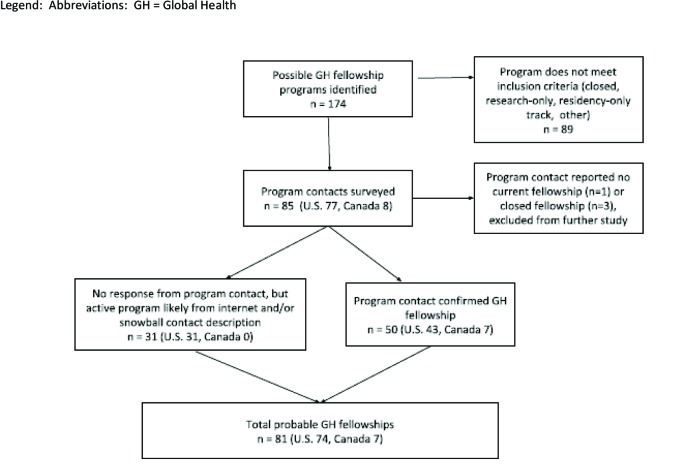
Flow diagram for identification of global health fellowship programs

**Figure s2.**
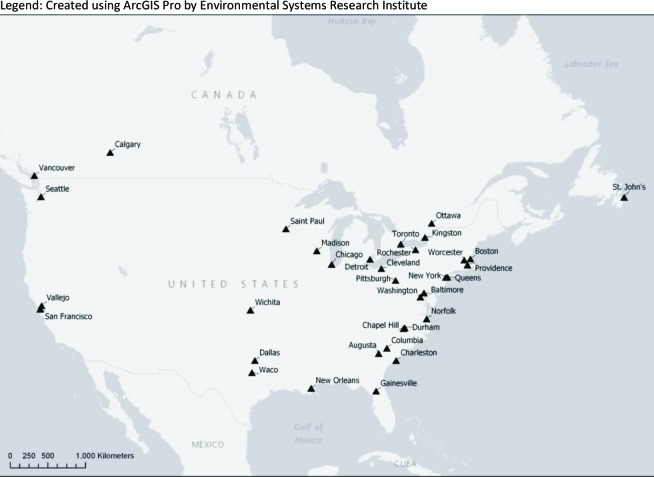
Map of identified GH fellowships in US and Canada, all specialties

## Appendix B

**Table s1:** Number of programs reporting fellowship activities by setting

	Clinical work (n=46)	Teaching (n=46)	Policy/Advocacy (n=40)	Coursework (n=46)	Research (n=46)
**Resource-limited, LMIC**	35 (76.1%)	42 (91.3%)	33 (82.5%)	7 (15.6%)	42 (91.3%)
**Resource-rich, North America**	34 (73.9%)	35 (76.1%)	25 (62.5%)	39 (86.7%)	21 (45.7%)
**Resource-limited, North America**	24 (52.2%)	14 (15.2%)	15 (37.5%)	7 (15.6%)	15 (32.6%)
**Resource-rich, LMIC**	3 (6.5%)	9 (19.6%)	10 (25.0%)	4 (8.9%)	10 (23.3%)

Abbreviations: LMIC: low- and middle-income countries

## Appendix C: Global health fellowship director survey

To better characterize current opportunities for trainees across disciplines, we are conducting this survey of global health fellowship programs available in the US and Canada.

We hope the results will be helpful to programs and trainees and thank you in advance for your participation.

**For this survey, “global health fellowship” is defined as formal training principally focused on global health beyond the minimum required length of training for residency. This additional period of training could be either subsequent to or integrated into residency training**.

1. Please enter the name of your institution

2. Does your academic department currently offer formal fellowship training in global health?

□ Yes

□ Not at this time (Skip to question 2a)

2a. Which of the following statements best describes your academic department’s history with global health fellowships?

□ We had a global health fellowship that has since been terminated (Skip to question 2b)

□ We have plans to begin a global health fellowship within the next two years (Skip to question 3).

□ We have never had a global health fellowship, and have no immediate plans to begin one (Thank-you for completing this survey)

2b. Please explain the circumstances around the closure of your global health fellowship program.

2c. Are you interested in participating in a future study addressing the topic of terminated global health fellowships?

□ Yes (Provide your contact information)

□ No (Thank-you for completing this survey)

3. From which clinical specialty (or specialties) does/will your global health fellowship accept applicants? Check all that apply.

□ Anesthesia

□ Emergency medicine

□ Family medicine

□ Internal medicine

□ Obstetrics and gynecology

□ Pediatrics

□ Surgery (any field)

□ Other (please specify)

4. Is your academic department located in the US or Canada?

□ US □ Canada

5. Please indicate the month and year in which your global health fellowship program was established.

Month: ____

Year: ______

6. When does your global health fellowship occur, relative to your residency program?

□ After residency training is completed (skip to question 6a)

□ Integrated with residency training (skip to question 6b)

□ Both options are available to our applicants (skip to question 6b)

6a. What is the typical length of your global health fellowship?

□ 6 months

□ 12 months

□ 18 months

□ 24 months

□ Other (please specify)

6b. What is the typical length of your global health fellowship (not including months devoted to other parts of residency training)?

□ 6 months

□ 12 months

□ 18 months

□ 24 months

□ Other (please specify)

7. How many global health fellowship positions do you typically offer each year?

8. Please estimate the number of global health fellows who graduated from your program between 2012 and 2016, inclusive? (Please do not include current fellows who have yet to graduate.)

9. What are the eligibility requirements for candidates to participate in your fellowship program? Please check all that apply.

□ Completed medical school

□ Completed residency training

□ Prior global health experience

□ Board eligibility

□ Other (please specify)

10. What criteria are most important in selecting your global health fellows? Please rank your responses from 1 to 6, where 1 = most important and 6 = least important.

□ Written application file (essays, CV, letters of recommendation, etc.)

□ Interview

□ Applicant’s previous global health experience

□ Applicant’s Masters of Public Health (MPH) or other advanced degree

□ Applicant’s intention to pursue global health as a major career focus

□ Applicant’s potential for leadership in global health

11. How is your fellowship funded (e.g., to pay for fellows’ salary, travel costs, coursework, administrative costs, etc)? Please check all that apply.

□ Fellow billing for patient care

□ Fellow self-funding

□ Department funds

□ Academic institutional grant

□ Private foundation

□ Public grant funding (e.g., NIH or CIHR)

□ Graduate medical education (GME) or government funding

□ Funds from international partner

□ Other (please specify)

**The next set of questions focuses on the content of your global health fellowship program**.

12. What best describes the role of each of the following activities in your global health fellowship?

**Table:**

	Mandatory	Optional	Not available
Coursework			
Clinical Work			
Research			
Teaching (by fellow)			
Policy/advocacy work			

13. Please describe any novel training/experiences related to any of these activities that are available to your fellows.

14. In which settings do your fellows complete the following activities?

**Table:**

	Resource-limited settings in North America	Resource-rich settings in North America	Resource-limited settings in low-or middle-income countries	Resource-rich settings in low-or middle-income countries
Coursework				
Clinical work				
Research				
Teaching (by fellow)				
Policy/advocacy work				

**The next few questions ask about what happens after fellows graduate from your program**.

15. Does your program formally follow up with your fellows regarding where they are working after graduation?

□ Yes

□ No

□ Not applicable (e.g., no graduates to date)

16. When following up with fellows, what outcomes are tracked (e.g., career activities and work setting)?

17. How are the outcomes measured (e.g., follow-up survey at 12 months post-graduation)?

18. What global health-related career activities do your fellows typically participate in after they graduate? Please check all that apply.

□ Advocacy

□ Direct patient care

□ Research

□ Policy development

□ Education

□ Administration

□ Other (please specify)

19. In 2016, what proportion (%) of your graduates to date spent at least 3 months of the year working in a low- or middle-income country after graduation?

□ I don’t know

Proportion: ____

**These last questions provide an opportunity for you to tell us more about your program**.

20. With which of the following organizations has your program established a partnership? Please check all that apply.

□ Non-governmental organizations (NGOs)

□ Medical schools/residency programs in low- and middle-income countries

□ Policy-makers or governments

□ Indigenous Band/Tribal Councils

□ Industry/private sector

□ We have not established partnerships with any of these organizations.

□ Other (please specify)

21. What components of your global health fellowship program have you cancelled or significantly changed because they were ineffective?

22. Please rank the following challenges in order of significance to your program, where 1 = most significant and 6 = least significant.

□ Lack of funding

□ Lack of experience global health faculty

□ Lack of political or institutional support

□ Lack of fellowship accreditation

□ Lack of qualified applicants

□ Lack of collaborating internal placement sites

23. Please tell us about other important challenges your program has faced that were not included in the previous question.

24. Please describe any aspects of your program that you consider innovative.

**Members of our team have established a public database of global health fellowship programs that aims to provide applicants and other stakeholders a current listing of global health fellowship programs in North America**.

25. May we include your global health fellowship in this database, listing your fellowship’s program title, city and state/province and website?

□ Yes

□ No

26. Please provide your fellowship program’s preferred website address.

27. We would like to ensure that we identify all global health fellowships in the U.S. and Canada.

Please list any global health fellowships of which you are aware (existing or in development) that are not already listed on our database. If possible, please include fellowship program title, location, academic institution, and/or any contact information you may have.

28. Thank you again for your willingness to complete this survey. If you have any additional comments or questions, please feel free to include them below.

29. If you would like us to email you a summary of the survey results, please provide your email address in the space provided.
